# Relationship between symptoms, barriers to care and healthcare utilisation among children under five in rural Mali

**DOI:** 10.1111/tmi.13592

**Published:** 2021-05-14

**Authors:** Emily Treleaven, Caroline Whidden, Faith Cole, Kassoum Kayentao, Mohamed Bana Traoré, Djoumé Diakité, Seydou Sidibé, Tracy Kuo Lin, David Boettiger, Souleymane Cissouma, Vincent Sanogo, Nancy Padian, Ari Johnson, Jenny Liu

**Affiliations:** ^1^ Institute for Social Research University of Michigan Ann Arbor MI USA; ^2^ Muso Bamako Mali; ^3^ Department of Disease Control London School of Hygiene and Tropical Medicine London UK; ^4^ Malaria Research & Training Centre University of Bamako Bamako Mali; ^5^ Institute for Health & Aging University of California San Francisco CA USA; ^6^ Kirby Institute University of New South Wales Sydney NSW Australia; ^7^ Mali Regional Health Directorate Bankass Mali; ^8^ Division of Prevention and Case Management National Malaria Control Programme Bamako Mali; ^9^ Department of Medicine University of California San Francisco CA USA

**Keywords:** child health, healthcare utilisation, barriers to care, Mali

## Abstract

**Objectives:**

To identify social and structural barriers to timely utilisation of qualified providers among children under five years in a high‐mortality setting, rural Mali and to analyse how utilisation varies by symptom manifestation.

**Methods:**

Using baseline household survey data from a cluster‐randomised trial, we assessed symptom patterns and healthcare trajectories of 5117 children whose mothers reported fever, diarrhoea, bloody stools, cough and/or fast breathing in the preceding two weeks. We examine associations between socio‐demographic factors, symptoms and utilisation outcomes in mixed‐effect logistic regressions.

**Results:**

Almost half of recently ill children reported multiple symptoms (46.2%). Over half (55.9%) received any treatment, while less than one‐quarter (21.7%) received care from a doctor, nurse, midwife, trained community health worker or pharmacist within 24 h of symptom onset. Distance to primary health facility, household wealth and maternal education were consistently associated with better utilisation outcomes. While children with potentially more severe symptoms such as fever and cough with fast breathing or diarrhoea with bloody stools were more likely to receive any care, they were no more likely than children with fever to receive timely care with a qualified provider.

**Conclusions:**

Even distances as short as 2–5 km significantly reduced children’s likelihood of utilising healthcare relative to those within 2 km of a facility. While children with symptoms indicative of pneumonia and malaria were more likely to receive any care, suggesting mothers and caregivers recognised potentially severe illness, multiple barriers to care contributed to delays and low utilisation of qualified providers, illustrating the need for improved consideration of barriers.

## Introduction

Under‐five mortality and morbidity remain unacceptably high in a number of countries despite investments to reduce barriers to care [1, 2, 3]. In Mali, the under‐five mortality rate was estimated as 101 per 1000 live births in 2018 [4]. Within the Malian health system, a pluralistic, fee‐for‐service system, patients face a number of barriers that inhibit or delay access to care, including user fees and long distances to primary and secondary health facilities [5, 6, 7]. Education and literacy; maternal decision‐making power, autonomy and control of resources; and language and ethnicity are important determinants of children’s healthcare utilisation [8, 9, 10]. Social disadvantages related to these characteristics may exacerbate structural barriers, further impeding children’s access to care.

To reduce the burden of morbidity and mortality, children require prompt access to quality care. Many of the leading causes of under‐five mortality in high‐burden settings are treatable, including pneumonia, diarrhoea and malaria [1], provided children are correctly diagnosed in a timely manner and receive effective treatment to avoid illness progression or death [11, 12, 13]. However, common symptoms indicative of potentially severe illness, such as fever and diarrhoea, can result from a diverse set of aetiologies [14]. In particular, aetiologies of febrile illnesses can be difficult to identify, resulting in delayed treatment or treatment with inappropriate drugs [14]. Compared to untrained and unqualified providers, doctors, nurses, midwives, trained community health workers (CHWs) and pharmacists are often better able to evaluate and diagnose a sick child, provide appropriate and effective treatments, and refer to higher level care when necessary [15, 16, 17, 18]. Yet, distance, cost and other barriers can deter patients from seeking care with a qualified provider, forcing them to delay treatment, turn to community‐based sources such as ambulatory drug sellers, or forego care altogether [5, 6, 7, 8, 9, 10, 11, 19]. Verbal and social autopsy data reveal that delayed entry to care with a qualified provider is a major contributor to preventable child death in Mali and other settings [13, 17].

Because common symptoms in early childhood may indicate a number of disease aetiologies requiring differential diagnosis and treatment, understanding how caregivers respond to specific symptoms is critical for informing the design of interventions that address the root causes of inequalities in access to care. When making decisions about when and where to seek care for a sick child, caregivers are informed by their prior experiences with childhood illness and the medical system [8, 20]. They also consider perceived severity, norms and beliefs related to specific symptoms and illnesses, and perceived quality of services [19, 20]. Caregivers must make decisions about how to most effectively invest limited resources to return a child to health, weighing cost, quality and distance against the perceived needs of the child. Thus, in addition to addressing key barriers such as cost and distance, effective interventions must also consider caregivers’ perceptions of which symptoms require prompt evaluation or treatment and which merit consultation with potentially costlier and/or harder‐to‐reach higher quality providers. Such a design will allow health systems to more effectively respond to the needs of the highest risk children.

In this paper, we have two objectives: (1) identify social and structural barriers to timely utilisation of qualified providers in response to symptoms of common childhood illnesses; (2) describe how the manifestation of specific symptoms and combinations of symptoms commonly observed in young children relates to timely utilisation of qualified providers. We examine these questions using detailed household survey data from Mali, including geolocated measures of distance, collected as part of the baseline round of a cluster‐randomised controlled trial on proactive case detection and management of common childhood illnesses.

## Methods

### *Study* *setting*

The data for this study were derived from the baseline survey of the Trial of Proactive Community Case Management to Reduce Child Mortality (NCT02694055) [21]. The objective of the trial is to assess the extent to which door‐to‐door proactive case detection for common childhood illnesses (e.g. diarrhoea, malaria, pneumonia and acute malnutrition) by CHWs can reduce under‐five mortality over a three‐year period compared to a conventional approach to the delivery of integrated community case management. The trial is being conducted in Bankass, a rural district of the Mopti region in eastern Mali, about 600 km east of Bamako. The Mopti region has particularly poor reproductive, maternal and child health indicators, with an under‐five mortality rate above the national average [4]. The study area is comprised of seven health catchment areas, each served by one public‐sector primary health centre (PHC), with a centralised public‐sector secondary referral hospital. Pharmacists, drug sellers and traditional healers are common sources of care within communities. At the time of the survey, there were about 55 CHWs operating in the area.

In this region, nuclear households are generally grouped together in family compounds based on familial ties. The head of the family compound is generally the eldest family member, male or female. Financial and social resources are often pooled at the compound level, and decisions related to children’s healthcare access and expenditures may be made at this higher level by the head of the family, or at the household level.

### *Study* *population and sample*

At baseline, 99 576 individuals in 15 839 households were enrolled across 137 village clusters. Village clusters were defined as a geographical grouping of homes at least one kilometre from the nearest geographical grouping of homes and could comprise a single village or hamlet, or several villages and/or hamlets. The sample included 16 393 children under five years of age whose mother completed a survey module on child health.

The analytic sample for the present study included children under age five who reported any illness in the two weeks preceding the survey and had complete information on socio‐demographic characteristics of interest, resulting in an analytic sample of 5117 children (62 children missing information on household wealth and 65 missing ethnicity were excluded). These children resided in 3795 unique households in 2433 unique family compounds.

### Data

The ProCCM trial baseline survey was conducted from December 2016 to January 2017. The survey instrument was adapted from the Demographic and Health Survey questionnaire and included a household roster and modules on household characteristics, administered to the female head of household or another household member at least 18 years of age. Eligible women of reproductive age (15 to 49 years) completed modules on contraceptive use, maternal health, lifetime birth history and healthcare utilisation for all co‐resident biological children under five years of age. Respondents provided information about children under five who experienced diarrhoea, fever and/or cough in the preceding two weeks, including the presence of blood in stools and fast breathing. Healthcare utilisation and treatment were reported separately for each symptom. Respondents were asked where and when care or advice was sought in relation to the onset of symptoms, as well as treatment(s) administered. All respondents provided written informed consent.

### Measures

Among all children under five, we examined the prevalence of diarrhoea (with or without bloody stools), febrile illness and cough (with or without fast breathing) within the two weeks preceding the survey. Diarrhoea was defined as three or more loose stools within 24 h. We coded symptom manifestations into the following mutually exclusive categories: diarrhoea, diarrhoea with bloody stools, fever, cough, cough with fast breathing, fever and diarrhoea, fever and diarrhoea with bloody stools, fever and cough, fever and cough with fast breathing, and diarrhoea and cough with or without fever (including bloody stools and/or fast breathing).

We examined four outcomes among children reporting an illness, representing utilisation behaviours that are increasingly likely to lead to optimal child health outcomes. Each outcome was coded as a binary variable according to the following definitions:

1. *Any care:* Child received any consultation, evaluation and/or treatment from any source, inside or outside the home, versus no consultation, evaluation or treatment;
2. *Care from a qualified provider:* Child received any consultation, evaluation and/or treatment inside or outside the home by a qualified provider (PHC, secondary hospital, CHW, pharmacy), versus any treatment, consultation or evaluation from an unqualified provider (drug shop or seller, traditional healer or other provider without medical training), where if a child received treatment from both qualified and unqualified providers, s/he was considered treated by a qualified provider;
3. *Care within 24 h:* child received any treatment within 24 h of symptom onset (i.e. the same day or one day after); and,
4. *Care from a qualified provider within 24 h:* child received treatment from a qualified provider within 24 h of symptom onset.

We included a number of covariates that represent social and structural barriers to care in regression analyses. These included indicators for mother’s ethnicity (Dogon, Peulh, other minority), educational attainment (any formal schooling, none) and decision‐making power. Women were coded as having any decision‐making power if they self‐reported that they either solely or jointly made decisions in any of three domains: regarding household purchases, visiting relatives or their own healthcare, using measures from the Demographic and Health Surveys [4]. Shared decision‐making could be joint with any other person. Household size was based on the household roster, which included household members who resided at the location more than half of the time. Using an index of ownership of durable goods, livestock and physical housing characteristics, we estimated relative wealth quintiles within the full trial sample using principle components analysis to generate asset scores for each household, replicating the procedure used by the Demographic and Health Surveys [4, 22]. Household distance to the nearest PHC was determined using orthodromic (great circle) distance estimates between family compound and PHC GPS coordinates, categorised as follows: <2 km, 2 to <5 km, 5 to <7 km, <7 to 10 km and >=10 km. We rounded categories to the nearest integer to facilitate interpretation. Households were considered to have clean water and improved sanitation in accordance with WHO standards [23]; we included these factors because they are markers of a household’s ability and willingness to invest in health. Finally, we controlled for child’s age and sex.

### Analysis

We first described the characteristics of children in the analytic sample, their symptoms and healthcare utilisation trajectories. We then conducted multilevel mixed‐effects regression analyses to assess the four utilisation outcomes for sick children. Regressions included random effects for family compound and village cluster; all other covariates were entered as fixed effects. Results were robust to alternate specifications of covariates. Analyses were performed using Stata version 16.1 (Stata Corporation, TX, USA).

### Ethical approval

The larger trial study (NCT026940550) received ethical approval from the Ethics Committee of the Faculty of Medicine, Pharmacy and Dentistry, University of Bamako.

## Results

**Figure 1:**
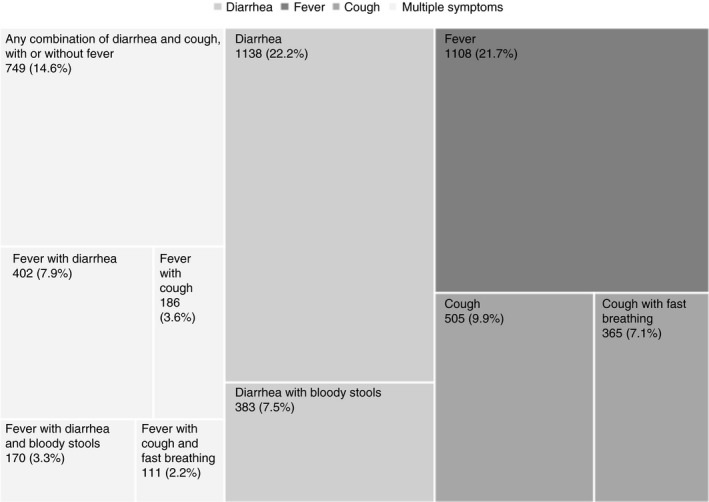
Illness symptoms reported among children under five in the two weeks preceding the survey (*N* = 5117).

The distribution of symptoms among 5117 children under five reporting at least one symptom of diarrhoea, fever or cough in the preceding two weeks is illustrated in Figure 1. Among the symptom combinations, diarrhoea was the most prevalent (1138; 22.2%), followed by fever (1108; 21.7%); 505 children reported cough (9.9%). Almost half (2366; 46.2%) of all children who reported any symptoms had more than one symptom (diarrhoea, blood in stools, fever, cough and fast breathing). For example, 383 children reported diarrhoea with bloody stools (7.5%), with or without fever, and 111 children (2.2%) reported cough with fast breathing, with or without fever.

Recently ill children had a median age of 31.0 months (IQR 17–46 months) with even distribution across sexes (Table 1). The ethnic composition and wealth distribution of the analytic sample was similar to the overall study population. Among these children, 91.7% had a mother who had no formal schooling, and 67.3% had a mother without any sole or shared household decision‐making power across the three domains. Households with recently ill children had a median of 6.0 members. Less than half of households had access to clean water (45.1%), and just over half had improved sanitation (53.1%). About one‐third of households were located within 5 km of the nearest PHC (34.7%), while 19.7% lived more than 10 km from the nearest PHC.

**Table 1:** Socio‐demographic characteristics of children under five years of age reported to have had any symptoms of diarrhoea, fever or cough in the last two weeks (*N* = 5117)

	*N* (%)
Child’s age	
0–5 months	362 (7.1)
6–11 months	458 (8.9)
12–23 months	962 (18.8)
24–35 months	1098 (21.5)
36–59 months	2237 (43.7)
Child’s sex	
Male	2557 (50.0)
Female	2560 (50.0)
Ethnicity	
Dogon	4638 (90.6)
Peulh	312 (6.1)
Other	167 (3.3)
Maternal education	
Mother has attended any school	424 (8.3)
Mother has no formal education	4693 (91.7)
Maternal decision‐making power	
Mother has decision‐making power in any domain	1674 (32.7)
Mother has no decision‐making power	3443 (67.3)
Household size	median 6.0
Household sanitation	
Improved	2398 (46.9)
Unimproved	2719 (53.1)
Household water source	
Improved	2808 (54.9)
Unimproved	2309 (45.1)
Household wealth quintile	
Poorest	1053 (20.6)
Poor	1029 (20.1)
Middle	1024 (20.0)
Rich	973 (19.0)
Richest	1038 (20.3)
Distance to nearest primary health centre (km)	
<2 km	637 (12.5)
2–<5 km	1137 (22.2)
5–<7 km	1331 (26.0)
7–<10 km	1003 (19.6)
>=10 km	1009 (19.7)

**Figure 2:**
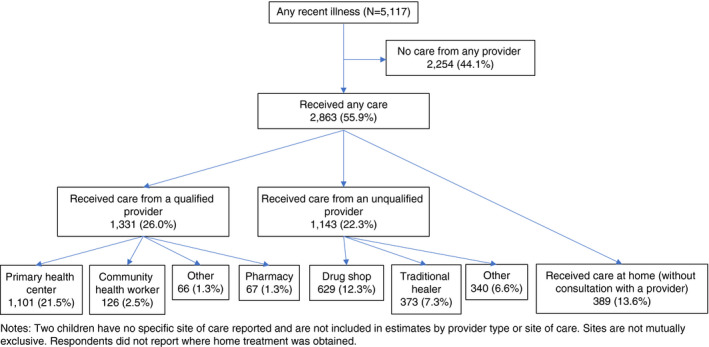
Trajectories of care among recently ill children under five years of (*N* = 5117).

Figure 2 presents children’s trajectories of care. Over half (55.9%) received any care (inside or outside the home), 26.0% received care from a qualified provider, and 22.3% received care from an unqualified provider. Among different sources of care, 21.5% received care at a PHC, 2.5% from a CHW, 1.3% at another public facility, and 1.3% at a pharmacy; 12.3% received care at a drug shop, 7.3% from a traditional healer, and 6.6% from someone else. Finally, 13.6% received treatment at home, not in consultation with a provider. Overall, 47.8% of children received care within 24 h, and 21.7% received care from a qualified provider within 24 h (data not shown).

Estimated effects for covariates of interest were relatively similar and consistent across regression models (Table 2). Distance from a health facility significantly predicted utilisation outcomes, even among children who resided relatively close to a PHC. Children who resided 2 to 5 km from the nearest PHC were half as likely to access care from a qualified provider within 24 h (aOR=0.53, 95% CI 0.34, 0.83) compared to those within 2 km. Children in the poorest wealth quintile had consistently lower odds of each utilisation outcome compared to the richest children; they had 0.47 times the odds of being seen by a qualified provider within 24 h (95% CI 0.34, 0.64). Children whose mothers had no formal education were significantly less likely to have each of the care outcomes met compared to children whose mothers had attended any school. Other significant factors included child’s age, improved sanitation, access to clean water and maternal decision‐making power. Younger children, especially those aged 6 to 11 months, were significantly less likely than newborns and infants 5 months of age or less to receive any care or care within 24 h.

**Table 2:** Adjusted odds of healthcare utilisation for children under five with recent illness (*N* = 5117)^†^

	Any care		Care from a qualified provider		Care within 24 h		Care from a qualified provider within 24 h	
	OR	95% CI	OR	95% CI	OR	95% CI	OR	95% CI
Child age								
0–5 months	(ref)	(ref)	(ref)	(ref)	(ref)	(ref)	(ref)	(ref)
6–11 months	0.64*	(0.43–0.95)	0.74	(0.49–1.11)	0.63*	(0.41–0.95)	0.68^+^	(0.44–1.06)
12–23 months	1.05	(0.74–1.49)	0.92	(0.65–1.31)	1.01	(0.70–1.45)	0.87	(0.59–1.27)
24–35 months	1.20	(0.86–1.69)	1.12	(0.79–1.58)	1.22	(0.86–1.74)	1.12	(0.77–1.61)
36–59 months	1.46*	(1.06–2.01)	1.25	(0.91–1.72)	1.32	(0.95–1.83)	1.14	(0.80–1.60)
Child is female	1.05	(0.90–1.24)	1.11	(0.95–1.30)	1.09	(0.92–1.27)	1.13	(0.95–1.33)
Maternal education								
No formal education	0.64**	(0.46–0.88)	0.67**	(0.50–0.89)	0.66*	(0.47–0.91)	0.68*	(0.50–0.92)
Attended any school	(ref)	(ref)	(ref)	(ref)	(ref)	(ref)	(ref)	(ref)
Maternal decision‐making power								
No decision‐making power	0.88	(0.72–1.09)	1.21^+^	(1.00–1.47)	0.77*	(0.62–0.95)	1.16	(0.95–1.44)
Decision‐making power in any domain	(ref)	(ref)	(ref)	(ref)	(ref)	(ref)	(ref)	(ref)
Ethnicity								
Dogon	(ref)	(ref)	(ref)	(ref)	(ref)	(ref)	(ref)	(ref)
Minority (Peulh, other)	0.69*	(0.48–0.99)	0.79	(0.56–1.10)	0.62*	(0.42–0.90)	0.84	(0.58–1.20)
Household size	0.99	(0.96–1.02)	1.01	(0.98–1.03)	0.98	(0.95–1.01)	1.00	(0.98–1.03)
Household wealth quintile								
Poorest	0.53***	(0.38–0.72)	0.56***	(0.42–0.75)	0.50***	(0.36–0.69)	0.47***	(0.34–0.64)
Poor	0.78	(0.57–1.05)	0.73*	(0.55–0.95)	0.81	(0.59–1.10)	0.74*	(0.55–0.98)
Middle	0.61**	(0.45–0.82)	0.62***	(0.47–0.81)	0.65**	(0.47–0.88)	0.60**	(0.45–0.80)
Rich	0.79	(0.58–1.06)	0.63**	(0.48–0.82)	0.82	(0.60–1.12)	0.64**	(0.48–0.85)
Richest	(ref)	(ref)	(ref)	(ref)	(ref)	(ref)	(ref)	(ref)
Household sanitation								
Unimproved	0.79*	(0.64–0.97)	0.64***	(0.53–0.77)	0.80*	(0.64–0.99)	0.68***	(0.55–0.83)
Improved	(ref)	(ref)	(ref)	(ref)	(ref)	(ref)	(ref)	(ref)
Household water source								
Unimproved	0.69**	(0.55–0.87)	0.90	(0.73–1.10)	0.63***	(0.49–0.80)	0.81^+^	(0.65–1.01)
Improved	(ref)	(ref)	(ref)	(ref)	(ref)	(ref)	(ref)	(ref)
Distance to nearest primary health centre								
<2 km	(ref)	(ref)	(ref)	(ref)	(ref)	(ref)	(ref)	(ref)
2–<5 km	0.56**	(0.37–0.85)	0.52**	(0.35–0.78)	0.64*	(0.42–0.97)	0.53**	(0.34–0.83)
5–<7 km	0.57*	(0.37–0.88)	0.38***	(0.25–0.58)	0.53**	(0.35–0.82)	0.37***	(0.23–0.60)
7–<10 km	0.44***	(0.28–0.68)	0.33***	(0.21–0.52)	0.46**	(0.29–0.72)	0.32***	(0.19–0.53)
10+ km	0.58*	(0.36–0.89)	0.40***	(0.25–0.64)	0.51**	(0.35–0.81)	0.40**	(0.24–0.68)

^†^ Controlling for health catchment area.

*** *P* < 0.001, **P < 0.01, *P < 0.05, ^+^ *P* < 0.10.

Controlling for relevant covariates (Table 3), children with symptoms indicative of pneumonia (i.e. fever and cough with fast breathing), diarrhoea with bloody stools, and fever and diarrhoea with bloody stools had the highest odds of receiving any care (aOR=2.12, 95% CI 1.13, 3.96; aOR=1.75, 95% CI 1.21, 2.52; aOR=1.64, 95% CI 0.99, 2.72, respectively). However, these children were not more likely than children with fever to receive timely care from a qualified provider. Children with diarrhoea, cough and fever with cough were significantly less likely than children with fever to experience each care outcome.

**Table 3:** Adjusted odds of healthcare utilisation for children under five with recent illness by symptom type (*N* = 5117)^†^

	Any care		Care from a qualified provider		Care within 24 h		Care from a qualified provider within 24 h	
	OR	95% CI	OR	95% CI	OR	95% CI	OR	95% CI
Symptom presentation								
Fever	(ref)	(ref)	(ref)	(ref)	(ref)	(ref)	(ref)	(ref)
Diarrhoea	0.55***	(0.43–0.71)	0.60***	(0.47–0.76)	0.52***	(0.40–0.67)	0.56***	(0.43–0.73)
Diarrhoea with bloody stools	1.75**	(1.21–2.52)	1.06	(0.77–1.47)	1.18	(0.82–1.69)	1.01	(0.71–1.42)
Cough	0.36***	(0.26–0.50)	0.35***	(0.25–0.49)	0.35***	(0.25–0.48)	0.36***	(0.25–0.52)
Cough with fast breathing	1.26	(0.87–1.82)	1.16	(0.84–1.62)	0.95	(0.66–1.38)	1.09	(0.76–1.55)
Fever and diarrhoea	0.72^+^	(0.51–1.02)	0.70*	(0.50–0.98)	0.77	(0.54–1.09)	0.71	(0.50–1.02)
Fever and diarrhoea with bloody stools	1.64^+^	(0.99–2.72)	1.48^+^	(0.95–2.30)	1.08	(0.65–1.78)	1.14	(0.70–1.86)
Fever and cough	0.39***	(0.25–0.61)	0.47**	(0.29–0.77)	0.40	(0.25–0.64)	0.40**	(0.24–0.69)
Fever and cough with fast breathing	2.12*	(1.13–3.96)	1.69^+^	(1.00–2.85)	1.45	(0.79–2.68)	1.32	(0.75–2.32)
Diarrhoea and cough with/without fever	0.79	(0.59–1.05)	0.88	(0.67–1.16)	0.70*	(0.52–0.94)	0.86	(0.64–1.14)

^†^ Controlling for age, sex, maternal education, maternal decision‐making power, ethnicity, wealth quintile, household size, clean water, sanitation, distance to health facility and health catchment area.

*** *P* < 0.001, **P < 0.01, *P < 0.05, ^+^ *P* < 0.10.

## Discussion

Our examination of social and structural barriers to care across diverse symptom combinations revealed that in rural Mali, a setting with a high burden of morbidity and mortality among children under five, just one‐fifth of children were treated by a qualified provider within 24 h of symptom onset, and almost half received no care at all. Poverty, greater distance to the nearest PHC, and a lack of maternal education were strongly and consistently associated with poorer utilisation of available healthcare services—representing key barriers to care in this setting. Children with multiple symptoms, including those that experienced symptoms indicative of malaria or pneumonia, tended to be more likely to receive any care than children with uncomplicated symptoms such as cough or diarrhoea, suggesting mothers and caregivers recognised potential danger signs. However, children with multiple symptoms such as fever and diarrhoea with blood in stools were not more likely than those with fever alone to receive timely care with a qualified provider. Low rates of prompt utilisation with qualified providers, particularly among children with potentially severe or life‐threatening symptoms such as blood in stools, highlight the need for improved triage and referral systems that directly address common barriers to care to ensure equitable access to diagnostic tools and effective case management, particularly for children most at risk of poor outcomes.

In this rural, disadvantaged setting, distance and poverty have widespread effects on care outcomes such that all but the closest and/or wealthiest children are significantly less likely to reach a qualified provider within 24 h, or even any care inside or outside the home. Rather than a linear relationship between distance and healthcare utilisation, we found children who resided 2–5 km from the nearest PHC had significantly lower odds of timely care with a qualified provider, similar to children residing five or more kilometres from a PHC. This suggests that even relatively short distances present substantial barriers to care. Children in the lowest three wealth quintiles were significantly disadvantaged in their likelihood of utilising qualified providers. When poverty is associated with utilisation of health services, this may indicate that both direct costs, such as user fees, transportation or accommodation, and/or indirect costs, such as time or opportunity costs, may have widespread effects as deterrents to care [5, 24]. Strategies that address these inter‐related barriers of distance and poverty together, such as community‐based services and the removal of user fees, may help to improve access to and utilisation of timely, qualified provider care [15, 25, 26, 27], even for those who live relatively close to a PHC. Notably, our findings regarding the effect of facility distance are at odds with national health policies in several countries, including Mali, Liberia and Benin, which currently deploy CHWs only in villages more than 5 km from the nearest health centre [28, 29, 30].

When comparing our study population to Mali’s population, 77.2% of our sample fell in the poorest wealth quintile relative to a nationally representative sample, and less than 5% of our sample were in Mali’s richest two quintiles [4]. Thus, the relative wealth differences between quintiles in this sample may be subtle, reflecting pervasive poverty in the area. That almost the entire sample falls in the poorer and middle wealth quintiles relative to a national sample may lead to an underestimate of the effect of wealth on healthcare utilisation. Our findings related to the effects of distance and wealth on care utilisation for children under five are consistent with previous studies. Across settings, wealthier households are more likely to seek any, timely and appropriate care [31, 32, 33, 34, 35, 36], and distance to a healthcare facility is inversely related to the frequency and timeliness of care utilisation [37, 38, 39].

While we found maternal education was a powerful indicator of higher likelihood of optimal child healthcare utilisation, we found weaker associations between maternal decision‐making power and utilisation outcomes. Maternal education is consistently associated with greater use of biomedical healthcare and reduced child mortality globally [40, 41, 42]. That decision‐making power was generally not associated with most outcomes in our analysis may be attributed to the relevance of the domains of decision‐making measured in our survey and whether they appropriately capture family dynamics related to child healthcare utilisation in this setting. Qualitative studies underscore the familial nature of decision‐making processes for child health in Mali, in which elder family members, especially males, tend to control the resources necessary to access healthcare [6, 7]. Our measure combined both sole and joint decision‐making power, which may not adequately capture the effects of maternal autonomy or control over resources in this setting, as very few women had sole decision‐making power. It may also be that determinants such as cost or distance are more powerful barriers to care. Addressing these barriers in addition to women’s education and decision‐making power may reduce delays to child healthcare with a qualified provider.

The majority of recently ill children in our sample experienced febrile illness or multiple symptoms, which may indicate a number of different aetiologies [14, 43]. Symptom patterns potentially indicative of more severe illness were generally associated with higher odds of utilising any care, though these differences were attenuated when examining children’s likelihood of timely care with a qualified provider. When caregivers recognise potential danger signs such as blood in stools, they may more sell productive assets or incur debt to overcome cost‐related barriers [7, 44]. However, even among children with the most concerning symptom patterns, only about one‐fourth received care from a qualified provider within 24 h. This underscores how common barriers to care are in this high‐burden setting. A study using verbal and social autopsy data from Mali found delays in reaching effective care were compounded by poor quality and other failures of the health system [18]. Thus, to fully address delays and gaps in care, effective interventions likely need to consider both demand and supply side factors, such as more rapid identification of children with potentially severe illness, reduction of distance to care, and improvement in the quality of services provided. For example, drawing on our findings related to distance and types of symptoms, a community health programme design whereby CHWs conduct regular and frequent proactive case‐finding home visits may further assist caregivers in accessing effective triage, diagnosis and treatment for sick children [45, 46, 47].

This study has several limitations. Symptoms and healthcare utilisation were self‐reported and may be subject to recall bias. We lacked data on the number of unique illness episodes a child experienced in the preceding two weeks, as well as reliable data on illness severity. We could not distinguish between low‐ and high‐grade fevers, for example. However, our data included symptoms generally considered as danger signs in infants and young children, and we were able to examine combinations of symptoms that generally indicate the need for prompt assessment and treatment by a trained provider, such as fever and cough with fast breathing. We accounted for non‐independence at the family compound level, which is a strength over household surveys such as the Demographic and Health Surveys, where this information is not available. Additionally, we used precise measures of family compound distance to PHCs.

Although children with symptom patterns indicative of more severe illness were more likely to have received any care, providing evidence that mothers and caregivers recognised potential danger signs, likelihood of timely care with a qualified provider did not vary by symptoms, and overall child healthcare utilisation was low in this context. In order to reduce under‐five morbidity and mortality, health systems should account for inter‐related barriers of distance, poverty and education by bringing qualified health services closer to communities and reducing direct and indirect costs, improving access to diagnostic tools and effective case management for children who need it most.

## References

[1] Liu L , Oza S , Hogan D *et al*. Global, regional, and national causes of under‐5 mortality in 2000–15: an updated systematic analysis with implications for the Sustainable Development Goals. Lancet 2016: 388: 3027–3035. 27839855 10.1016/S0140-6736(16)31593-8 PMC5161777

[2] Black RE . Progress in the use of ORS and zinc for the treatment of childhood diarrhea. J Glob Health 2019: 9: 10101. 10.7189/09.010101 PMC6344068 30701067

[3] McAllister DA , Liu L , Shi T *et al*. Global, regional, and national estimates of pneumonia morbidity and mortality in children younger than 5 years between 2000 and 2015: a systematic analysis. Lancet Glob Health 2019: 7: e47–e57. 30497986 10.1016/S2214-109X(18)30408-X PMC6293057

[4] Institut National de la Statistique (INSTAT), Cellule de Planification et de Statistique Secteur Santé‐Développement Social et Promotion de la Famille (CPS/SS‐DS‐PF), ICF . Enquête Démographique et de Santé au Mali 2018. Bamako, Mali, et Rockville, Maryland, USA: INSTAT, CPS/SS‐DS‐PF et ICF; 2019.

[5] Johnson A , Goss A , Beckerman J , Castro A . Hidden costs: the direct and indirect impact of user fees on access to malaria treatment and primary care in Mali. Soc Sci Med 2012: 75: 1786–1792. 22883255 10.1016/j.socscimed.2012.07.015

[6] Ellis AA , Traore S , Doumbia S , Dalglish SL , Winch PJ . Treatment actions and treatment failure: case studies in the response to severe childhood febrile illness in Mali. BMC Public Health 2012: 12: 946. 23127128 10.1186/1471-2458-12-946 PMC3497867

[7] Ellis AA , Doumbia S , Traoré S , Dalglish SL , Winch PJ . Household roles and care‐seeking behaviours in response to severe childhood illness in Mali. J Biosoc Sci 2013: 45: 743–759. 23601075 10.1017/S0021932013000163

[8] Colvin CJ , Smith HJ , Swartz A *et al*. Understanding careseeking for child illness in sub‐Saharan Africa: A systematic review and conceptual framework based on qualitative research of household recognition and response to child diarrhoea, pneumonia and malaria. Soc Sci Med 2013: 86: 66–78. 23608095 10.1016/j.socscimed.2013.02.031

[9] Geldsetzer P , Williams TC , Kirolos A *et al*. The recognition of and care seeking behaviour for childhood illness in developing countries: a systematic review. PLoS One 2014: 9: e93427. 24718483 10.1371/journal.pone.0093427 PMC3981715

[10] Ellis AA , Winch P , Daou Z , Gilroy KE , Swedberg E . Home management of childhood diarrhoea in southern Mali—Implications for the introduction of zinc treatment. Soc Sci Med 2007: 64: 701–712. 17097788 10.1016/j.socscimed.2006.10.011

[11] Kallander K , Hildenwall H , Waiswa P , Galiwango E , Peterson S , Pariyo G . Delayed care seeking for fatal pneumonia in children aged under five years in Uganda: a case‐series study. Bull World Health Organ 2008: 86: 332–338. 18545734 10.2471/BLT.07.049353 PMC2647445

[12] Waiswa P , Kallander K , Peterson S , Tomson G , Pariyo GW . Using the three delays model to understand why newborn babies die in eastern Uganda. Trop Med Int Health 2010: 15: 964–972. 20636527 10.1111/j.1365-3156.2010.02557.x

[13] Snavely ME , Maze MJ , Muiruri C *et al*. Sociocultural and health system factors associated with mortality among febrile inpatients in Tanzania: a prospective social biopsy cohort study. BMJ Glob Health 2018: 3: e000507. 10.1136/bmjgh-2017-000507 PMC5841511 29527339

[14] D’Acremont V , Kilowoko M , Kyungu E *et al*. Beyond malaria — causes of fever in outpatient Tanzanian children. N Engl J Med 2014: 370: 809–817. 24571753 10.1056/NEJMoa1214482

[15] Das JK , Lassi ZS , Salam RA , Bhutta ZA . Effect of community based interventions on childhood diarrhea and pneumonia: uptake of treatment modalities and impact on mortality. BMC Public Health 2013: 13: S29. 24564451 10.1186/1471-2458-13-S3-S29 PMC3953053

[16] Treleaven E , Liu J , Prach LM , Isiguzo C . Management of paediatric illnesses by patent and proprietary medicine vendors in Nigeria. Malaria Journal 2015: 14: 1–9. 26041654 10.1186/s12936-015-0747-7 PMC4465720

[17] Willcox ML , Kumbakumba E , Diallo D *et al*. Circumstances of child deaths in Mali and Uganda: a community‐based confidential enquiry. Lancet Glob Health 2018: 6: e691–e702. 29773123 10.1016/S2214-109X(18)30215-8

[18] Sazawal S , Black RE . Effect of pneumonia case management on mortality in neonates, infants, and preschool children: a meta‐analysis of community‐based trials. Lancet Infect Dis. 2003: 3: 547–556. 12954560 10.1016/s1473-3099(03)00737-0

[19] Scott K , McMahon S , Yumkella F , Diaz T , George A . Navigating multiple options and social relationships in plural health systems: a qualitative study exploring healthcare seeking for sick children in Sierra Leone. Health Policy Plan 2014: 29: 292–301. 23535712 10.1093/heapol/czt016

[20] Leonard KL . Active patients in rural African health care: implications for research and policy. Health Policy Plan 2014: 29: 85–95. 23307907 10.1093/heapol/czs137

[21] Whidden C , Treleaven E , Liu J *et al*. Proactive community case management and child survival: protocol for a cluster randomised controlled trial. BMJ Open 2019: 9: e027487. 10.1136/bmjopen-2018-027487 PMC6720240 31455700

[22] Filmer D , Pritchett LH . Estimating wealth effects without expenditure Data—Or tears: an application to educational enrollments in states of India. Demography 2001: 38: 115–132. 11227840 10.1353/dem.2001.0003

[23] World Health Organization, UNICEF , others. Core questions on drinking water and sanitation for household surveys. 2006 (Available from: http://apps.who.int/iris/handle/10665/43489) [6 Mar 2017].

[24] Rutherford ME , Mulholland K , Hill PC . How access to health care relates to under‐five mortality in sub‐Saharan Africa: systematic review: access to health care and child mortality. Trop Med Int Health 2010: 15: 508–519. 20345556 10.1111/j.1365-3156.2010.02497.x

[25] Kalyango JN , Alfven T , Peterson S , Mugenyi K , Karamagi C , Rutebemberwa E . Integrated community case management of malaria and pneumonia increases prompt and appropriate treatment for pneumonia symptoms in children under five years in Eastern Uganda. Malar J 2013: 12: 340. 24053172 10.1186/1475-2875-12-340 PMC3848942

[26] Rutebemberwa E , Kadobera D , Katureebe S , Kalyango JN , Mworozi E , Pariyo G . Use of community health workers for management of malaria and pneumonia in urban and rural areas in Eastern Uganda. Am J Trop Med Hyg. 2012: 87(5 Suppl): 30–35. 23136275 10.4269/ajtmh.2012.11-0732 PMC3748519

[27] Mubiru D , Byabasheija R , Bwanika JB *et al*. Evaluation of integrated community case management in eight districts of Central Uganda. PLoS One 2015: 10: e0134767. 26267141 10.1371/journal.pone.0134767 PMC4534192

[28] White EE , Downey J , Sathananthan V *et al*. A community health worker intervention to increase childhood disease treatment coverage in rural Liberia: a controlled before‐and‐after evaluation. Am J Public Health 2018: 108: 1252–1259. 30024811 10.2105/AJPH.2018.304555 PMC6085010

[29] Ministere de la Sante et de l’Hygiene Publique . Soins essentiels dans la communaute: Guide de mise en oeuvre. Direction Nationale de la Sante: Bamako, Mali, 2015.

[30] Devlin K , Egan KF , Pandit‐Rajani T . Community Health Systems Catalog Country Profile: Benin. Advancing Partners & Communities: Arlington, Virginia, 2017, 18.

[31] Burton DC , Flannery B , Onyango B *et al*. Healthcare‐seeking behaviour for common infectious disease‐related illnesses in rural Kenya: a community‐based house‐to‐house survey. J Health Popul Nutr 2011: 29: 61–70. 21528791 10.3329/jhpn.v29i1.7567 PMC3075057

[32] Rutebemberwa E , Kallander K , Tomson G , Peterson S , Pariyo G . Determinants of delay in care‐seeking for febrile children in eastern Uganda. Trop Med Int Health 2009: 14: 472–479. 19222823 10.1111/j.1365-3156.2009.02237.x

[33] Deressa W , Ali A , Berhane Y . Household and socioeconomic factors associated with childhood febrile illnesses and treatment seeking behaviour in an area of epidemic malaria in rural Ethiopia. Trans R Soc Trop Med Hyg 2007: 101: 939–947. 17602716 10.1016/j.trstmh.2007.04.018

[34] Sultana M , Sarker AR , Sheikh N *et al*. Prevalence, determinants and health care‐seeking behavior of childhood acute respiratory tract infections in Bangladesh. PLoS One 2019: 14: e0210433. 30629689 10.1371/journal.pone.0210433 PMC6328134

[35] Volpicelli K , Buttenheim AM . Do social factors predict appropriate treatment of child diarrheal disease in Peru? Matern Child Health J 2016: 20: 2299–2308. 27449783 10.1007/s10995-016-2049-2

[36] Ayalneh AA , Fetene DM , Lee TJ . Inequalities in health care utilization for common childhood illnesses in Ethiopia: evidence from the 2011 Ethiopian Demographic and Health Survey. Int J Equity Health 2017: 16: 67. 28431502 10.1186/s12939-017-0561-7 PMC5399816

[37] Gombojav N , Manaseki‐Holland S , Pollock J , Henderson AJ . The effects of social variables on symptom recognition and medical care seeking behaviour for acute respiratory infections in infants in urban Mongolia. Arch Dis Child 2009: 94: 849. 19574234 10.1136/adc.2008.157115

[38] Müller O , Traoré C , Becher H , Kouyaté B . Malaria morbidity, treatment‐seeking behaviour, and mortality in a cohort of young children in rural Burkina Faso. Trop Med Int Health 2003: 8: 290–296. 12667146 10.1046/j.1365-3156.2003.01030.x

[39] Sarrassat S , Meda N , Badolo H , Ouedraogo M , Somé H , Cousens S . Distance to care, care seeking and child mortality in rural Burkina Faso: findings from a population‐based cross‐sectional survey. Trop Med Int Health 2019: 24: 31–42. 30347129 10.1111/tmi.13170 PMC6378618

[40] Mensch BS , Chuang EK , Melnikas AJ , Psaki SR . Evidence for causal links between education and maternal and child health: systematic review. Trop Med Int Health 2019: 24: 504–522. 30767343 10.1111/tmi.13218 PMC6519047

[41] Frost MB , Forste R , Haas DW . Maternal education and child nutritional status in Bolivia: finding the links. Soc Sci Med 2005: 60: 395–407. 15522494 10.1016/j.socscimed.2004.05.010

[42] Bicego GT , Boerma JT . Maternal education and child survival: a comparative study of survey data from 17 countries. Soc Sci Med 1993: 36: 1207–1227. 8511650 10.1016/0277-9536(93)90241-u

[43] Kiemde F , Spijker R , Mens PF , Tinto H , Boele M , Schallig HDFH . Etiologies of non‐malaria febrile episodes in children under 5 years in sub‐Saharan Africa. Trop Med Int Health 2016: 21: 943–955. 27159214 10.1111/tmi.12722

[44] Dasré A , Samuel O , Hertrich V . The dynamics of the family network during childhood: a genealogical and longitudinal approach to rural Mali. Demogr Res 2019: 41: 231–262.

[45] Whidden C , Thwing J , Gutman J *et al*. Proactive case detection of common childhood illnesses by community health workers: a systematic review. BMJ Glob Health 2019: 4: e001799. 10.1136/bmjgh-2019-001799 PMC6936477 31908858

[46] Winch PJ . Intervention models for the management of children with signs of pneumonia or malaria by community health workers. Health Policy Plan 2005: 20: 199–212. 15965032 10.1093/heapol/czi027

[47] Theodoratou E , Al‐Jilaihawi S , Woodward F *et al*. The effect of case management on childhood pneumonia mortality in developing countries. Int J Epidemiol 2010: 39(Suppl 1): i155–i171. 20348118 10.1093/ije/dyq032 PMC2845871

